# The Critical Role of IL-34 in Osteoclastogenesis

**DOI:** 10.1371/journal.pone.0018689

**Published:** 2011-04-08

**Authors:** Zhi Chen, Kalman Buki, Jukka Vääräniemi, Guoliang Gu, H. Kalervo Väänänen

**Affiliations:** 1 Department of Cell Biology and Anatomy, Institute of Biomedicine, University of Turku, Turku, Finland; 2 University of Eastern Finland, Kuopio campus, Kuopio, Finland; University of Western Ontario, Canada

## Abstract

It has been widely believed that the cytokines required for osteoclast formation are M-CSF (also known as CSF-1) and RANKL. Recently, a novel cytokine, designated IL-34, has been identified as another ligand of CSF1R. This study was to explore the biological function, specifically osteoclastogenesis and bone metabolism, of the new cytokine. We produced recombinant mouse IL-34 and found that together with RANKL it induces the formation of osteoclasts both from splenocytes as well as dose-dependently from bone marrow cells in mouse and these cells also revealed bone resorption activity. It also promotes osteoclast differentiation from human peripheral blood mononucleated cells. Finally, we show that systemic administration of IL-34 to mice increases the proportion of CD11b+ cells and reduces trabecular bone mass. Our data indicate that IL-34 is another important player in osteoclastogenesis and thus may have a role in bone diseases. Strategies of targeting CSF1/CSF1R have been developed and some of them are already in preclinical and clinical studies for treatment of inflammatory diseases. Our results strongly suggest the need to revisit these strategies as they may provide a new potential pharmaceutical target for the regulation of bone metabolism in addition to their role in the treatment of inflammatory diseases.

## Introduction

Osteoclasts are multinucleated giant cells which have the capacity to resorb bone. They are derived from the hematopoietic progenitor of the myeloid lineage by a cytokine-driven proliferation and differentiation process. Since the identification of the receptor activator of NFκB ligand (RANKL) as the key regulator for osteoclast differentiation [1], for a decade, it has been believed that the cytokines required for osteoclast formation are macrophage colony-stimulating factor (M-CSF, also known as CSF-1) and RANKL [1], [2]. These factors are produced primarily by bone marrow stromal cells, osteoblasts and activated T cells [3]. RANK is a member of a family of proteins known as the tumor necrosis factor receptors and is expressed in osteoclasts and their precursors. The role of RANKL in osteoclastogenesis and bone resorption has been well documented in recent years [1], [4]–[6]. M-CSF deficient mice showed osteopetrosis due to severe deficiency of osteoclasts and macrophages [7], [8]. The osteoclast formation and bone resorption defects observed in M-CSF deficient mice were rescued by systemic administration of M-CSF [8], [9]. The crucial role of M-CSF on osteoclastogenesis was further supported by the study on the naturally occurring ‘toothless’ mutation in rat which was found to be due to the mutation of the Csf1 (M-CSF) gene [10].

In recent years, M-CSF or RANKL-independent osteoclastogenesis has also been noted. In the presence of TNF-α and TGF-β, an *in vitro* culture of hematopoietic precursors from RANKL-, RANK-, or TRAF6-deficient mice can differentiate to osteoclasts, suggesting the potential existence of alternative routes for osteoclast differentiation [11]. Systemic TNF-α increased the number of osteoclast precursors in circulation [12]. Further studies demonstrated that TNF-α upregulated the expression of c-Fms (Csf1r), IL-1 and IL-1R in bone marrow [13], [14]. Both IL-1 and TNF are inflammatory cytokines mediating bone resorption in a variety of diseases affecting bone. IL-1 has not only been shown to enhance the expression of RANKL in bone marrow stromal cells, therefore inducing osteoclast formation, but through the IL-1/IL-1R signaling, it also has the potential to induce osteoclastogenesis which is RANK/RANKL independent [15], [16].

M-CSF is a key cytokine for the development of macrophage lineage from hemopoietic stem cells and it is also required for the development of microglia. However, the microglia in the brains of adult M-CSF deficient mice developed normally, suggesting the existence of another factor that can substitute for the effect of M-CSF on this cell type [17]. The effect of M-CSF on osteoclast differentiation is mediated by its receptor, CSF1R. Similar to CSF-1 mutation *Csf1^op^/Csf1^op^* mice, deficiency of CSF1R also resulted in osteopetrosis, reduced mononuclear phagocyte and reproductive defect indicating the function of CSF-1 is through CSF1R. However, more severe phenotypes including osteopetrosis in these mice have also been observed, suggesting the existence of alternative factor(s) sharing the same receptor [18].

Recently, functional screening of a library of secreted proteins after transfection of an embryonic kidney cell line with recombinant cDNAs resulted in identification of a novel cytokine, designated IL-34 [19]. The novel cytokine was shown to stimulate the viability of monocytes and colony formation of macrophages from bone marrow cells. By screening of extracellular domains of transmembrane proteins, the receptor of IL-34 was discovered, and was found to be a known receptor, CSF1R [19].

To assess the role of the new cytokine, IL-34, in the process of osteoclast differentiation, we produced recombinant IL-34 in our lab. In this study we found that IL-34 together with RANKL induces the formation of osteoclasts both from splenocytes as well as from bone marrow cells in mouse in a dose-dependent manner and these cells also have bone resorption activity. In human, it also promotes the osteoclast differentiation from peripheral blood mononucleated cells. Finally, we show that systematic administration of IL-34 to mouse increases the number of CD11b+ cells and reduces bone mass. Thus, our data point to another important player for osteoclastogenesis and bone metabolism.

## Results

### IL-34 in combination with RANKL can induce mouse osteoclast differentiation both from bone marrow cells as well as from splenocytes

We first generated and purified recombinant murine IL-34 in our lab, (Figure S1a). Peptide sequence analysis showed that sequences from our purified protein correlated with the sequence of mouse IL-34 (Figure S1b).

**Figure 1:**
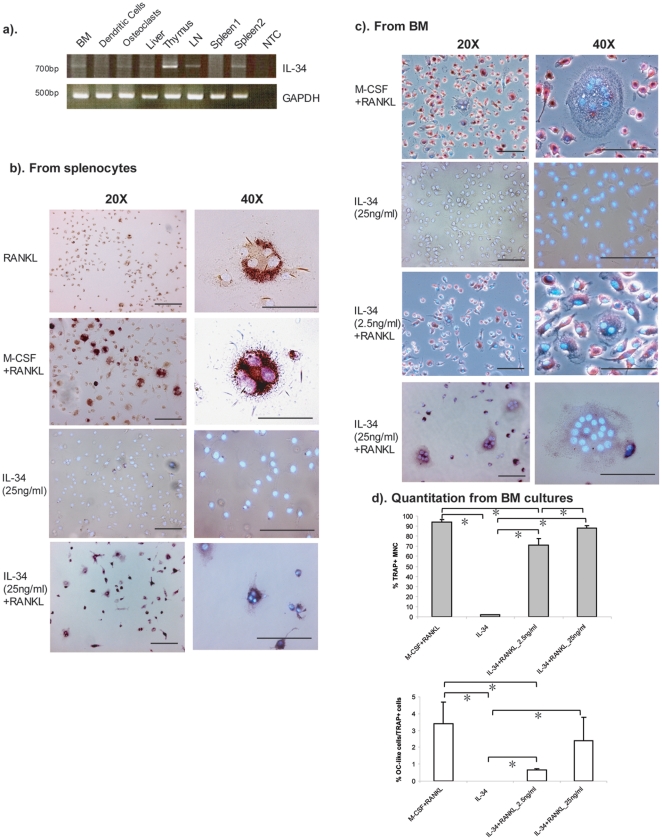
IL-34 combined with RANKL promotes the differentiation of mouse osteoclast-like cells from splenocytes and bone marrow. (a). The expression of Il34 in mouse tissues was performed by RT-PCR. Total RNA from mouse tissues were isolated. BM: bone marrow. LN: lymph nodes. NTC: no template control. (b). Splenocytes were isolated from 6–8-week-old Balb/c mice and cultured for 9 days in the presence of RANKL (100 ng/ml) alone, IL-34 (25 ng/ml) alone or RANKL combined either with 25 ng/ml of M-CSF or with 25 ng/ml of IL-34. The cells were fixed with 3% paraformaldehyde and were subjected to TRAP and Hoechst 33258 staining. (c). Bone marrow cells were isolated from the femurs and tibias of 6–8-week-old Balb/c mice. After depletion of adherent stromal cells by culturing the cells overnight with α-MEM media, the nonadherent bone marrow cells were cultured for 9 days in the presence of 25 ng/ml of IL-34 alone, 25 ng/mL recombinant M-CSF or with a different concentration of rmIL-34 (2.5 ng/ml, 25 ng/ml) and 100 ng/ml of RANKL. Three independent experiments were performed. All images in this study were acquired by Leica DMRB microscope and Leica DC300F digital camera system. Representative images are shown with a magnification of 20× or 40×. Bars, 100 µm. (d). The number of TRAP^+^ mononuclear cells and TRAP^+^ multinucleared cells (≥3 nuclei, shown as OC-like cells) were counted under microscopy. Data shown were average number counted from four wells. One-way ANOVA analysis was performed and was followed by Turkey's and Dunnett's post-hoc test by using SPSS statistic analysis software. *: The mean difference is significant at the 0.05 level.

As Lin et al. [19] demonstrated that IL-34 is highly expressed in spleen, we first wanted to know if it is also expressed in other lymphoid tissues. According to our RT-PCR results, IL-34 was detected in samples from mouse thymus, lymph nodes, spleen, as well as bone marrow and liver (Figure 1a). Previous studies have shown that RANKL, which is another key factor for osteoclastogenesis, can be produced by activated lymphocytes [4], [20]. IL-34 can specifically bind to CSF1R [19], this led us to speculate on its possible overlapping effect with M-CSF. We first wanted to test the possibility that in combination with RANKL, IL-34 induces the differentiation of osteoclasts in lymphoid tissues. When splenocytes were cultured with exogenous RANKL alone for nine days, most of the TRAP positive cells were mononuclear small cells, and only few TRAP positive binuclear or multinuclear cells were observed. The addition of M-CSF and RANKL increased the amount of multicleated TRAP positive osteoclast-like cells as already shown previously [1]. Addition of IL-34 alone, cells proliferated and attached, but majority of the cells were TRAP negative mononuclear cells (Figure 1b). However, not only was the cell proliferation increased by addition of IL-34 combined with RANKL, but also the number of multinuclear TRAP positive osteoclast-like cells was increased (Figure 1b). With the same concentration (25 ng/ml), the effect of IL-34 is comparable with M-CSF.

If IL-34 has the similar function as M-CSF on osteoclast differentiation from splenocytes, is it able to induce osteoclastogenesis from bone marrow cells? To test this, we cultured bone marrow derived non-adherent cells for nine days with or without RANKL and different concentrations of IL-34. Again, without exogenous RANKL, IL-34 alone could not induce the formation of TRAP positive multinuclear cells (Figure 1c). IL-34 had an effect on cell proliferation as the density of cells was much lower when IL-34 was added at a lower concentration (2.5 ng/ml) (Figure 1c). Combined with RANKL, the addition of IL-34 increased the number of TRAP positive multinucleated osteoclast-like cells. Furthermore, this effect was dose-dependent (Figure 1c, 1d). These results demonstrate that IL-34, similar to M-CSF (CSF1), combined with RANKL induces the formation of TRAP positive multinucleated osteoclast-like cells both from splenocytes as well as from bone marrow cells.

### *In vitro* differentiated osteoclasts by IL-34 and RANKL show dose-dependent bone resorption activity

**Figure 2:**
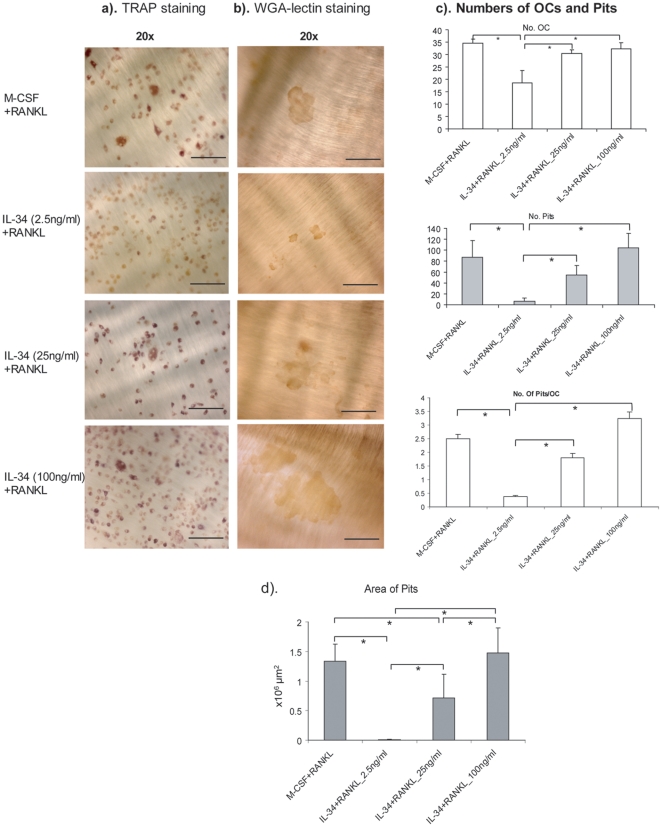
*In vitro* differentiated osteoclasts by IL-34 and RANKL show dose-dependent bone resorption activity. Mouse nonadherent bone marrow cells were cultured on bone slices for 9 days in the presence of RANKL and M-CSF or RANKL with 2.5 ng/ml, 25 ng/ml, 100 ng/ml of IL-34. The bone slices with cells were fixed and stained for TRAP, and all TRAP-positive multinucleated cells were counted and analyzed under a microscope. The cells were removed followed by WGA-lectin staining for pits. Subsequent counting of resorption pits was performed with a microscope. Representative images of TRAP staining (a) and WGA-lectin staining (b) under different culturing conditions. Representative images are shown with a maginification of 20×. Bars, 100 µm. (c). Histogram of number of osteoclast-like cells, number of pits under different culturing conditions and number of pits/osteoclast (n = 5). (d). Area of Pits was quantitated using an Olympus microscope connected to a computer and the OsteoMeasure program (version 3.21; OsteoMetrics, Atlanta, GA, USA), n = 5. One-way ANOVA analysis was performed and was followed by Turkey's and Dunnett's post-hoc test by using SPSS statistic analysis software. *: The mean difference is significant at the 0.05 level.

In order to test whether in vitro differentiated osteoclast-like cells by IL-34 and RANKL are functional, we cultured bone marrow derived non-adherent cells on bone slices for 9 days with the above described conditions followed by TRAP staining and WGA-lectin staining for pits. As shown in Figure 2, as a control, M-CSF (25 ng/ml) plus RANKL induced the differentiation of TRAP positive cells and the formation of pits. With the increased concentration of exogenous IL-34, the number of TRAP positive cells was also increased. Moreover, the number of pits formed as well as the size of the pits also increased with the dose of IL-34, indicating that in vitro differentiated osteoclasts by IL-34 and RANKL have bone resorption activity.

### *In vitro* differentiation of human osteoclasts by IL-34 and RANKL

**Figure 3:**
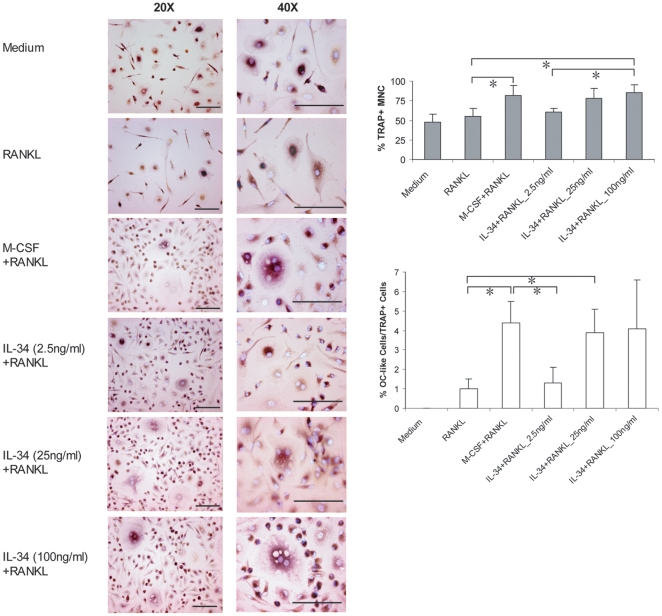
*In vitro* differentiation of human osteoclasts by IL-34 and RANKL. CD14^+^ human mononuclear cells were isolated from human peripheral blood followed by purification with anti-CD14 coated meganetic beads. The cells were cultured with RANKL alone or RANKL combined either with M-CSF or with human IL-34 at the indicated concentrations for 9 days. Cells were fixed followed by TRAP and Hoechst 33258 staining. Three independent experiments were performed. Representative images are shown with a maginification of 20× and 40×. Bars, 100 µm. The right panel showed the quantitation of the number of TRAP+ mononuclear cells and TRAP+ multinucleared cells (≥3 nuclei, shown as OC-like cells) counted under microscopy. Data shown were average number counted from four wells. One-way ANOVA analysis was performed and was followed by Turkey's and Dunnett's post-hoc test by using SPSS statistic analysis software. *: The mean difference is significant at the 0.05 level.

To extend our findings beyond the species barrier, we performed our in vitro osteoclast differentiation experiment with human cells. Mononuclear cells were isolated from human peripheral blood and were further purified with anti-CD14 coated magnetic beads. These CD14+ human mononuclear cells were cultured with human IL-34 and RANKL for 9 days. A similar effect to that of IL-34 on human osteoclast differentiation was also observed. Since the experiment was started with human CD14+ mononuclear cells, many of them were positive by TRAP staining. However, very few multinucleated TRAP positive cells were observed. This situation was not changed by the addition of RANKL alone (Figure 3). As expected, M-CSF plus RANKL induced formation of multinucleated TRAP positive giant cells, which was served as a positive control of the experiment. The addition of IL-34 clearly caused the proliferation of human CD14+ mononuclear cells, which was indicated as increased cell density as well as the increased number of TRAP+ cells even at a very low concentration. Formation of multinucleated giant osteoclast-like cells was clearly observed with the increased concentration of exogenous IL-34 (Figure 3). The results are similar to what we observed in mouse cells, suggesting that the significant role of IL-34 in osteoclast differentiation is not species-specific. Furthermore, this effect is found on both splenocytes as well as bone marrow derived cells.

### Osteoblasts are one of the cellular sources of IL-34

**Figure 4:**
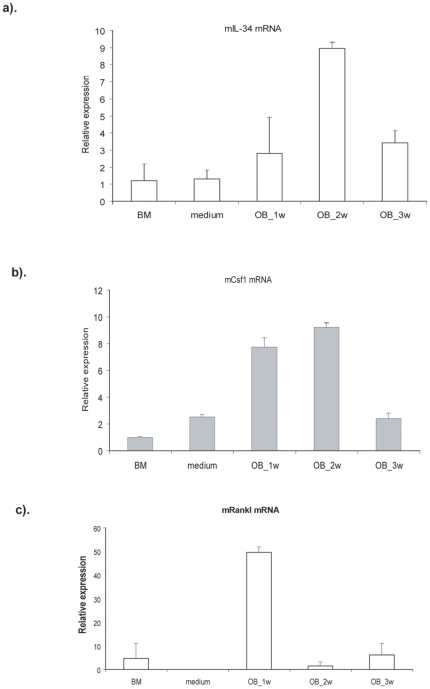
IL-34 is expressed by osteoblasts. Bone marrow cells were cultured in phenol red-free α-MEM media supplemented with 10% fetal calf serum, l0 nmol/L dexamethasone, 50 µg/mL ascorbic acid, and 10 mmol/L sodium β-glycerophosphate. Cells were harvested after 1, 2 and 3 weeks culture and total RNA was isolated. The expression of Rankl, Csf1 and Il34 were detected by real-time quantitative RT-PCR. Hprt was used as an endogenous control. Three independent experiments were performed.

The literature [19] and our results (Figure 1) indicate that IL-34 is highly expressed in the spleen and combined with RANKL, it has the capacity to induce osteoclast differentiation from splenocytes and bone marrow derived cells. The next step was to discover the cellular sources of IL-34 in bone. Therefore, we cultured bone marrow derived cells towards as osteoblast lineage for three weeks and kinetically detected the expression of IL-34 at the mRNA level. Although IL-34 was expressed at a low level in bone marrow cells, its expression was significantly induced during osteogenesis and peaked at 2 weeks (Figure 4a). Interestingly, M-CSF (CSF1), a cytokine with a similar function to IL-34, also showed a similar expression pattern to IL-34 (Figure 4b). It is known that osteoblasts can produce RANKL [21]. Our results indicated that the expression of RANKL by osteoblasts showed different kinetics that peaked at one week and then decreased (Figure 4c).

### Systemic administration of IL-34 to mice reduces bone mass

**Figure 5:**
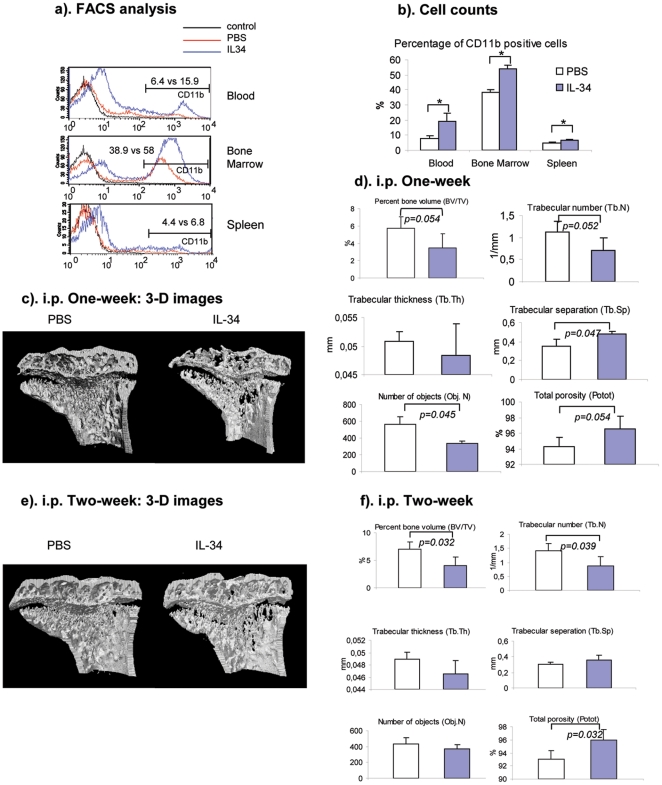
Systemic administration of IL-34 to mice increases the number of monocytes/macrophages and reduces bone mass *in vivo*. rmIL-34 was injected into 8-week old Balb/c mice daily at 250 ug/kg, i.p. After one week or two weeks of injections, the mice were sacrificed. Cells from the peripheral blood, bone marrow and spleen were treated with ACK buffer to lyse red blood cells, followed by anti-CD11b-PE staining. CD11b-positive cells were detected by FACSCalibur. (a). Representative FACS histogram images from CD11b-PE staining from peripheral blood, bone marrow cells and splenocytes. (b). Cell numbers of CD11b-positive cells in peripheral blood, bone marrow and spleen from PBS or IL-34 injected mice (n = 4). * indicating p<0.05. (c). Representative 3-D micro-CT images of the metaphyseal region of proximal tibias from mice injected with PBS or IL-34 for one week. (d). Histograms of 3-D trabecular structure parameters from micro-CT analysis (n = 3). (e). Representative 3-D micro-CT images of the metaphyseal region of proximal tibias from mice injected with PBS or IL-34 for two weeks. (f). Histograms of 3-D trabecular structure parameters from micro-CT analysis (n = 4).

To further characterize the role of IL-34 on osteoclast differentiation and bone resorption in vivo, mrIL-34 was injected into Balb/c mice intraperitonealy. After two weeks of injection, the proportion of CD11b+ cells from bone marrow, spleen and peripheral blood was significantly increased (Figure 5a, 5b), suggesting the critical role of IL-34 in monocyte/macrophage lineage differentiation and proliferation. To determine whether the increased number of macrophages, including osteoclasts leads to more active bone resorption, the phenotype of bone from IL-34 injected mice was studied by micro-CT. After one week of injections, a decreased bone mass of the proximal tibias from IL-34 injected mice was observed when compared to mice injected with PBS (Figure 5c). The decreased bone mass was also indicated by a reduced percentage of bone volume, trabecular number and increased trabecular separation and total porosity (Figure 5d). From a longer period of injection, 2 weeks, the decreased trabecular density in IL-34 injected mice was not only again observed from 3-D reconstruction of the micro-CT images compared with PBS injected mice (Figure 5e), but also indicated by the significantly changed micro-CT derived 3-D trabecular structure parameters of the proximal tibias of IL-34 injected mice (Figure 5f). The data from in vivo study strongly support the role of IL-34 for osteoclast differentiation and bone resorption.

## Discussion

Our study showed that IL-34 can replace M-CSF for osteoclast differentiation both in mouse and human. This provides experimental evidence supporting IL-34 as another ligand of CSF1R. However, despite the studies showing the effect of IL-34 on monocyte proliferation [19] and osteoclast differentiation, the function of this new cytokine is still largely unknown. As our data show that systemic administration of IL-34 increases CD11b+ cells, it is therefore important to explore whether it can induce the differentiation of macrophages or monocytes in other tissues. Previous studies have suggested the critical role of RANKL in osteoclast differentiation. We have also shown that neither RANKL nor IL-34 alone can induce osteoclast formation suggesting IL-34 is necessary but not sufficient (data not included).

Based on current very limited knowledge about this new cytokine, another important issue need to be noted is that IL-34 is highly expressed in spleen both in mouse and human. Giving the crucial function of spleen in immune responses, it is also worth to explore the biological function of IL-34 in spleen or as an extension, in immune responses. Now it seems clear that IL-34 has essential role in myeloid differentiation and proliferation. Myeloid cells are critical for innate and adaptive immune responses. Obviously the next question would be what its function is as an immuno-stimulant? Study how it interacts with lymphocytes will help us to understand its role during infection and inflammation.

M-CSF is produced by macrophages, monocytes, and stromal cells. What are the cellular sources of IL-34? We show in this study that IL-34 is expressed by osteoblasts. Bone structure and integrity are maintained through bone remodeling, a continuous process of bone resorption and deposition, which is coordinated through the relative activities of osteoblasts and osteoclasts. The theory developed by Rodan and Martin [22] suggested that osteoblasts are somehow able to instruct osteoclasts to resorb bone matrix, and therefore determine both the catabolic and anabolic phase of remodeling. After resorption is finished, the surface of the remaining bone attracts osteoblasts, possibly by releasing growth factors from the matrix [23], a process called coupling. Our data showed that IL-34 can regulate osteoclast formation and IL-34 is highly expressed by osteoblasts. Previous studies have shown that osteoblasts can produce RANKL and M-CSF, another two key cytokines for osteoclast differentiation. Now the third player for osteoclastogenesis has been identified and can also be produced by the same cell type indicating that osteoblasts are not only a bone forming cell, but also play an important regulatory role in bone homeostasis in the hematopoietic stem cell niche by producing these cytokines to coordinate the differentiation process of bone resorbing osteoclasts.

It has been described that two different isoforms of IL-34 exist. The two isoforms differ by an additional glutamine (Q) in isoform 1, which is the first one identified and designed as IL-34 [19]. We cloned the isoform 1 that has been proven by DNA sequencing of the plasmid (data not shown in this manuscript). In Figure S1, the two protein bands on gel (J1 and J2) have been sequenced and J1 correlates with isoform 1 (with an extra Q meaning glutamine, highlighted in yellow in figure S1). Mass spectra of trypsin-digested J2 shows that it is correlated with the same protein. We do not know exactly how the J2 is generated, possibly truncated by a nonspecific Pichia protease in the fermentation broth or it may be underglycosylated. J2 is a much smaller band and gives very low amount of protein. We used gelfiltration to purify the recombinant protein and that reduced the minor product to a minimum. Therefore, the majority of the purified protein is from J1 which has been used in this study. The other two highlighted letters in the figure S1 (NIT & NAT) are the consensus N-glycosylation sites that are usually glycosylated by eukaryotic cells (that includes Pichia pastoris). Glycosylation makes the cloned protein band spread on SDS-PAGE. The substantial differences between both recombinant protein and the cloned IL-34 both on the C- and N-terminal domains are due to that we left out the original signal sequence of the protein, which are the first 20 amino acids; instead, we used the Pichia signal sequence built into the plasmid, that would be surely recognized by the yeast. We also changed C-terminal for isolation purposes: a small added sequence with 6 histidines at the end for metal chelate affinity chromatography.

M-CSF has been recognized as a critical factor stimulating the formation of monocyte/macrophage lineage from pluripotent hematopoietic stem cells [24]. It is not only a primary regulator of the survival, proliferation and function of this cell lineage but also plays an important role in the pathogenesis of various diseases including bone diseases, inflammatory diseases and cancer. Therefore, efforts towards targeting M-CSF or M-CSFR signaling have been made by several pharmaceutical companies [25]. However, identification of the new cytokine, IL-34 which shares the same receptor with M-CSF and our present data indicate that targeting of M-CSF alone is not sufficient to block the effect through CSF1R. Very little is known about the biological significance of this new ligand of CSF1R, but it appears that the concept of targeting CSF1/CSF1R signaling may need to be revisited. RANKL or CSF1 signaling have been the target of clinical trials for the treatment of osteoporosis and autoimmune inflammatory diseases. Our data suggest that IL-34 may also be a potential pharmaceutical target for the treatment of bone and inflammatory diseases.

While preparing this manuscript, another two studies have recently been published. Baud'huin et al. showed that IL-34 is highly expressed by giant cell tumours of bone and by using in vitro culture, IL-34 is important in RANKL-induced osteoclastogenesis [26]. By putting IL-34 under Csf1 promoter, Wei et al. generated a transgenic mouse model to compare the functions of CSF-1 and IL-34 in regulation of myeloid cells. They also showed the bone phenotype of Csf1^op/op^ mouse was rescued by this transgenic mouse [27] [29]. Both of these two studies suggested that IL-34 plays important role in regulating osteoclastogenesis. Our study specifically focuses on osteoclastogenesis both from human and mouse progenitors. We showed here that combined with RANKL, IL-34 not only induced the formation of osteoclast, but also formed osteoclasts that had bone resorbing activity. We further show the systematic administration of IL-34 increases the number of monocytes and reduces the bone mass in vivo. Therefore, our results and data link the role of IL-34 directly to bone physiology and opens new possibilities to potential clinical applications.

## Materials and Methods

### Cloning and production of recombinant mouse IL-34 (rmIL-34)

Mouse IL34 reading frame minus signal sequence was PCR amplified from mouse spleen cDNA with the following primers: forward - ggtGAATTCaacgagaatttggagatatggac, reverse – GGTtctagaCCGGGCAACGAGCCATGGCTT. The PCR product (665 bp) was cloned into the pPICZαA vector (Invitrogen). Isolated clones were sequenced and proved to be isoform 1, containing one extra gutamine residue. The construct was electroporated into Pichia pastoris, strain X33 and integrated constructs were selected on Zeocin-yeast extract-peptone-glucose agar plates. Zeocine-resistant clones were picked.

A 10 mL inoculum was started from a colony overnight. It was inoculated into 800 mL buffered glycerol-complex medium containing 1% yeast extract 2% peptone, 100 mM potassium phasphate pH6, 1.34% yeast nitrogen base, 4×10−5% biotin, and 1% glycerol.

After vigorous overnight shaking at 30°C the cells were harvested by centrifugation and resuspended in the same medium containing 0.5% methanol in place of glycerol and shaken again for 24 h. Then, the fermentation broth was centrifuged to obtain a clear supernatant. Ni-NTA–agarose beads (5 mL settled volume) were mixed in and gently rotated in cold conditions for 1 h. Then the beads were separated and filled into a small chromatography column, washed with 50 mM phosphate buffer containing 300 mM NaCl and 15 mM imidazole, pH 7.8. The bound protein was eluted with 250 mM imidazole in the same buffer. Recombinant IL-34 was further purified by gelfiltration on a sephacryl S100 column. A small amount of purified IL-34 was run on 10% SDS-PAGE followed by silver staining. The protein bands on gel were Trypsin digested and then were proceeded to mass spectrometry analysis.

### *In vitro* differentiation of osteoclasts

Splenocytes were isolated from 6–8-week-old Balb/c mice. Bone marrow cells were also isolated from the femurs and tibias of 6–8-week-old Balb/c mice bred in the Central Animal Laboratory of the University of Turku. By culturing the cells overnight with α-MEM media (Gibco, New York, NY), adherent stromal cells were depleted These nonadherent bone marrow cells were cultured for 9 days in the presence of 25 ng/mL recombinant M-CSF (M-CSF) (R&D Systems, Minneapolis, MN) or with a different concentration of rmIL-34 and RANKL (100 ng/ml, Peprotech, UK).

For the human experiments, human peripheral blood mononuclear cells were isolated from the peripheral blood of healthy donors by Ficoll-Paque Plus (Amersham Pharmacia Biotech, Uppsala, Sweden). CD14^+^ monocytes were purified using CD14^+^ antibody-coated microbeads (Miltenyi Biotec, Bergisch Gladbach, Germany) according to the manufacturer's instructions. Purified CD14^+^ cells were cultured in the presence of 25 ng/mL M-CSF (M-CSF) (R&D Systems, Minneapolis, MN) or with a different concentration of recombinant human IL-34 (R&D Systems, Minneapolis, MN) and RANKL.

After 9 days in culture, the cells were fixed with 3% paraformaldehyde and were subjected to tartrate resistant acid phosphatase (TRAP) staining with kit 387-A (Sigma, St Louis, MO) as well as Hoechst 33258 (Molecular Probes, Eugene, USA) staining, according to the manufacturer's instructions.

### Bone resorption assay

After a 9-day culture, bone slices with cells were fixed with 3% paraformaldehyde and 2% sucrose in PBS for 10 min at room temperature. The slices were stained for TRAP as has been described previously [28], and all TRAP-positive multinucleated cells were counted and analyzed under a microscope. Quantification of resorption pits was performed according to Selander et al. [29]. The cells were removed by wiping the surface of the slices with a soft brush. The bone slices were then incubated with peroxidase-conjugated WGA-lectin (Sigma, St. Louis, MO) and diluted 1∶40 in PBS for 45 min. The bone slices were then washed with PBS, incubated in DAB solution (3,3′-diaminobenzidine tetrahydrochloride, 0.52 mg/ml in PBS containing 0.03% H_2_O_2_) for 5–10 min, and rinsed in PBS. Subsequent counting of resorption pits was performed with microscope. The area resorbed was quantitated using an Olympus microscope connected to a computer and the OsteoMeasure program (version 3.21; OsteoMetrics, Atlanta, GA, USA).

### *In vitro* differentiation of osteoblasts

Bone marrow cells were obtained from the femurs of 8- week-old female Balb/c mice and non-adherent cells were removed. All cultures were carried out in phenol red-free α-MEM media supplemented with 10% fetal calf serum (Bioclear UK, Wilts, UK), l0 nmol/L dexamethasone (Sigma, St. Louis, MO), ascorbic acid (50 µg/mL), 10 mmol/L sodium β-glycerophosphate, and antibiotics in 5% CO_2_, at 37°C. Cells were harvested after 1, 2 and 3 weeks culture and total RNA was isolated from these cells.

### Real-time quantitative polymerase chain reaction (TaqMan) analysis

Total RNA was isolated with an RNeasy kit (Qiagen, Valencia, CA). Complementary DNA was synthesized with the use of a TaqMan Reverse Transcription kit (Applied Biosystems, Foster City, CA) using random hexamers as primers according to the manufacturer's instructions. Hypoxanthine guanine phosphoribosyltransferase (Hprt) was used as an endogenous control. TaqMan primers and probes for mouse Csf1, Rankl, Il34 and Hprt were purchased from Applied Biosystems, and samples were analyzed using the ABI Prism 7900 Sequence Detection System (Applied Biosystems).

### Systemic administration of IL-34, FACS and micro-CT analysis

250 ug/kg recombinant murine IL-34 was daily injected intraperitonealy to 8-week old Balb/c mice bred in the Central Animal Laboratory of the University of Turku. The animal experiments were reviewed and approved by the local Ethics Committee on Animal Experimentation at the University of Turku and by the local Provincial State Office of Western Finland. After one-week or two-week injections, the mice were sacrificed. Cells from the peripheral blood, bone marrow and spleen were treated with ACK buffer to lyze red blood cells followed by anti-CD11b-PE (BD Bioscience, San Diego, CA) staining. Cells were detected and analysed by FACSCalibur (BD Bioscience, San Jose, CA).

Trabecular bone morphometry within the metaphyseal region of the proximal tibia was quantified using micro-CT (SkyScan1174, SkyScan, Belgium). Volumetric regions for trabecular analysis were selected within the endosteal borders to exclude the growth plate. Trabecular morphometry was characterized by measuring the bone volume fraction (bone volume / total volume, BV/TV), trabecular thickness (Tb. Th) and trabecular number (Tb. N). Image analysis was performed using the program “CT-analyser” and the “CT-volume” program (both programs are from SkyScan, Belgium) for 3D visualization of scanned objects.

### Statistical analysis

All error bars on graphs show means + SD. One-way ANOVA analysis was performed and was followed by Turkey's and Dunnett's post-hoc test by using SPSS statistic analysis software. The mean difference is significant at the 0.05 level. Student's *t test* was used to compare the micro-CT data generated from PBS or IL-34 injected mice, which was shown in Figure 5.

## Supporting Information

Figure S1 **Expression and production of recombinant mouse IL-34.** (**a**). Mouse IL-34 reading frame was amplified by PCR from mouse spleen cDNA and cloned into the pPICZαA vector. The construct was electroporated into Pichia pastoris, strain X33 and expressed protein was purified and run on 10% SDS-PAGE followed by Coomassie blue staining (left) and silver staining (right). The protein bands on silver stained gel were Trypsin digested and proceeded to mass spectrometry analysis. (**b**). Peptide sequences from mass spectrometry analysis (J1) compared with the expected sequence of mouse IL-34 (mil34cloned). Red letters are identified residues from J1 band. The other two highlighted letters (NIT & NAT) are the consensus N-glycosylation sites that are usually glycosylated by eukaryotic cells.
(TIF)
