# Supplementary figures and images for: The Critical Role of IL-34 in Osteoclastogenesis
Source: PLoS One. 2011 Apr 8;6(4):e18689. doi: 10.1371/journal.pone.0018689 (PMC3072988; doi:10.1371/journal.pone.0018689)

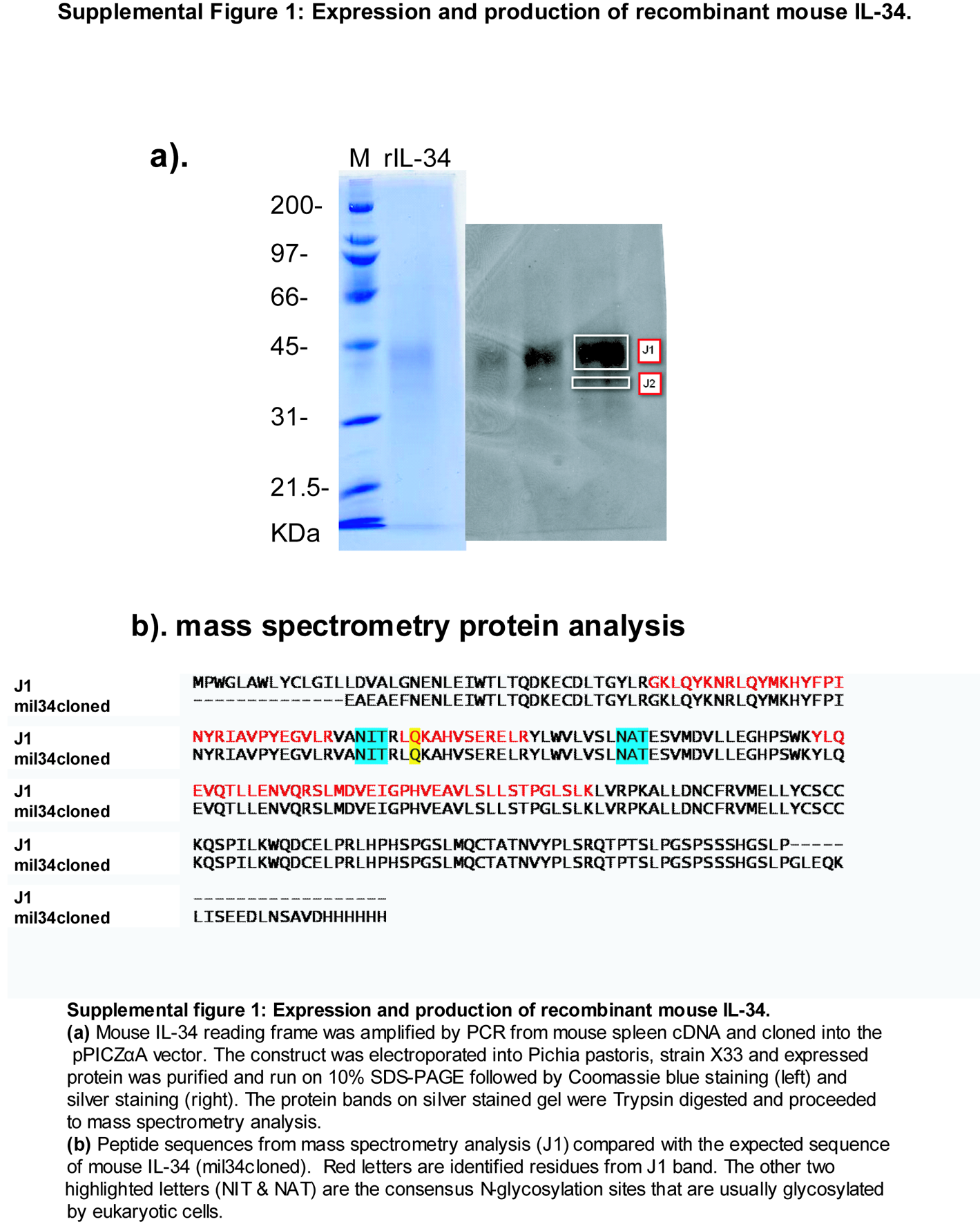

Supplement: Figure S1 — Expression and production of recombinant mouse IL-34. (a). Mouse IL-34 reading frame was amplified by PCR from mouse spleen cDNA and cloned into the pPICZαA vector. The construct was electroporated into Pichia pastoris, strain X33 and expressed protein was purified and run on 10% SDS-PAGE followed by Coomassie blue staining (left) and silver staining (right). The protein bands on silver stained gel were Trypsin digested and proceeded to mass spectrometry analysis. (b). Peptide sequences from mass spectrometry analysis (J1) compared with the expected sequence of mouse IL-34 (mil34cloned). Red letters are identified residues from J1 band. The other two highlighted letters (NIT & NAT) are the consensus N-glycosylation sites that are usually glycosylated by eukaryotic cells. (TIF) [file pone.0018689.s001.tif]
